# The Complex Role of Gut Microbiota in Systemic Lupus Erythematosus and Lupus Nephritis: From Pathogenetic Factor to Therapeutic Target

**DOI:** 10.3390/microorganisms13020445

**Published:** 2025-02-18

**Authors:** Emanuele Parodi, Marialuisa Novi, Paolo Bottino, Edoardo La Porta, Guido Merlotti, Luigi Mario Castello, Franca Gotta, Andrea Rocchetti, Marco Quaglia

**Affiliations:** 1 Nephrology and Dialysis Unit, SS. Antonio e Biagio e Cesare Arrigo University Hospital, 15121 Alessandria, Italy; emanuele.parodi@ospedale.al.it; 2 Gastroenterology Unit, SS. Antonio e Biagio e Cesare Arrigo University Hospital, 15121 Alessandria, Italy; mnovi@ospedale.al.it; 3 Microbiology Unit, SS. Antonio e Biagio e Cesare Arrigo University Hospital, 15121 Alessandria, Italy; fgotta@ospedale.al.it (F.G.); arocchetti@ospedale.al.it (A.R.); 4 Nephrology and Dialysis Unit, IRCCS Istituto Giannina Gaslini, 16147 Genoa, Italy; edoardolaporta@gaslini.org; 5 Department of Primary Care, Azienda Socio Sanitaria Territoriale (ASST) of Pavia, 27100 Pavia, Italy; gmerlotti87@gmail.com; 6 Internal Medicine Unit, SS. Antonio e Biagio e Cesare Arrigo University Hospital, 15121 Alessandria, Italy; luigi.castello@med.uniupo.it; 7 Department of Translational Medicine, Università del Piemonte Orientale (UPO), 28100 Novara, Italy

**Keywords:** gut microbiota, dysbiosis, autoimmunity, systemic lupus erythematosus, lupus nephritis, probiotic, fecal microbiota transplant

## Abstract

The role of gut microbiota (GM) and intestinal dysbiosis in triggering the onset and/or modulating the severity and progression of lupus nephritis (LN) has been the object of intense research over the last few years. Some alterations at the phyla level, such as the abundance of Proteobacteria and reduction in Firmicutes/Bacteroidetes (F/B) ratio and in α-diversity have been consistently reported in systemic lupus erythematosus (SLE), whereas a more specific role has been ascribed to some species (*Bacteroides thetaiotaomicron* and *Ruminococcus gnavus*) in LN. Underlying mechanisms include microbial translocation through a “leaky gut” and subsequent molecular mimicry, immune dysregulation (alteration of IFNγ levels and of balance between Treg and Th17 subsets), and epigenetic interactions. Levels of bacterial metabolites, such as butyrate and other short-chain fatty acids (SCFAs), appear to play a key role in modulating LN. Beyond bacterial components of GM, virome and mycobiome are also increasingly recognized as important players in the modulation of an immune response. On the other hand, microbiota-based therapy appears promising and includes diet, prebiotics, probiotics, symbiotics, and fecal microbiota transplantation (FMT). The modulation of microbiota could correct critical alterations, such as F/B ratio and Treg/Th17 imbalance, and blunt production of autoantibodies and renal damage. Despite current limits, GM is emerging as a powerful environmental factor that could be harnessed to interfere with key mechanisms leading to SLE, preventing flares and organ damage, including LN. The aim of this review is to provide a state-of-the-art analysis of the role of GM in triggering and modulating SLE and LN on the one hand, while exploring possible therapeutic manipulation of GM to control the disease on the other hand.

## 1. Introduction

**Figure 1:**
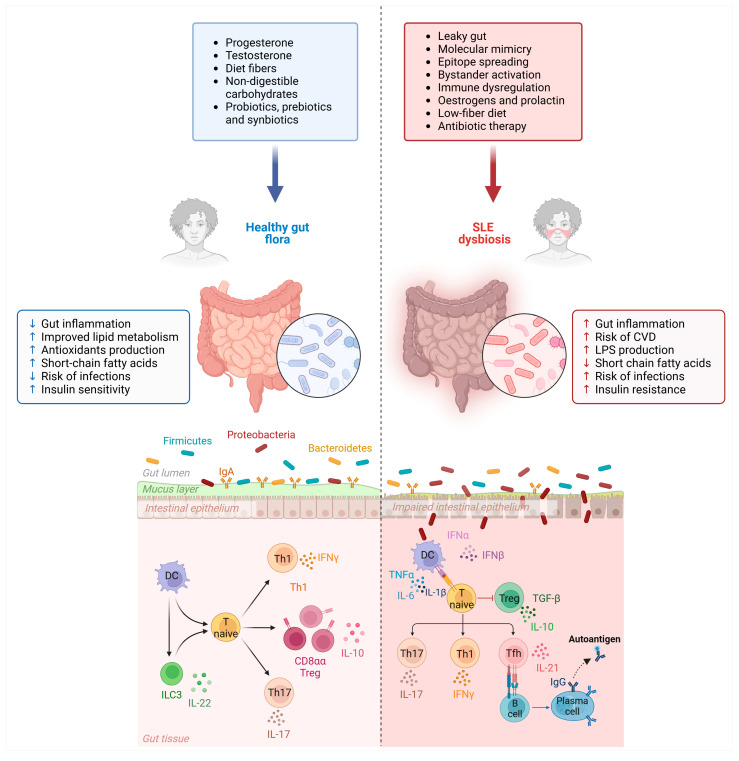
Modulators and actions of gut microbiota (GM) in healthy conditions and in systemic lupus erythematosus (SLE) (created in BioRender. Merlotti, G. (2025) https://BioRender.com/c97z713 (accessed on 12 February 2025)).

Gut microbiota (GM) plays a key role in maintaining gut barrier integrity and immune homeostasis, thus preventing the expansion and potentially mucosal invasion of harmful species [1] and exerting multiple beneficial functions (Figure 1).

The term dysbiosis denotes the disruption or imbalance in the composition and functions of GM, leading to an unhealthy state [2]. This imbalance can be due to changes in diversity, abundance, and distribution of microbial species within the gut ecosystem [3]. Several studies have shown a correlation between gut dysbiosis and inflammatory and autoimmune disorders (AIDs) such as type 1 diabetes, multiple sclerosis, rheumatoid arthritis and also systemic lupus erythematosus (SLE) [4]. A growing body of evidence indicates that overgrowth, or bloom, of commensal gut bacteria or pathobionts and relative reduction in symbiotic bacteria can shift a physiological balance, alter microbiome–host relationship and eventually lead to increased permeability of the gut epithelial barrier, creating a “leaky gut syndrome” (LGS) [5]. This condition can trigger abnormal immune responses to microbial antigens [6] and predispose to the development of SLE and lupus nephritis (LN) in genetically susceptible patients [7] (Figure 1)

## 2. Aim and Methods

The aim of this review is to provide a state-of-the-art analysis of the role of GM in triggering and modulating SLE and LN on the one hand while exploring possible therapeutic manipulation of GM to control the disease on the other hand.

PubMed library was searched from inception to November 2024, using a combination of Medical Subject Headings (MeSH) and keywords related to SLE and GM, including GM, dysbiosis, LGS, autoimmunity, SLE, LN, probiotic, prebiotic, and fecal microbiota transplant (FMT). References for relevant articles have also been checked.

## 3. GM Alterations in SLE and LN

GM has been increasingly recognized as an important factor in triggering SLE or exacerbating the disease [8], acting as a key environmental factor that interacts with the immune system and genetic predisposition [9]. Several studies have shown that transferring microbiota from lupus-prone mice into healthy ones could induce the disease, and germ-free mice receiving fecal matter from SLE mice develop increased levels of circulating anti-dsDNA antibodies [8]. Longitudinal gut microbiome analysis has shown a correlation between expansions of specific bacterial populations and clinical flares [10]. Differences in several aspects of GM have been demonstrated in SLE, both in animal models and in patients. These include reduced diversity, altered composition, aberrant activation of metabolic pathways and instability over time [11].

### 3.1. Reduced GM Diversity

Studies of fecal samples from SLE patients with active disease have also shown lower diversity compared to healthy controls [11]. Notably, Azzouz et al. documented that a decrease in microbiota α-diversity was inversely correlated with the SLE Disease Activity Index (SLEDAI) [12]. A recent systematic review by A Wang et al. also confirmed a significant reduction in diversity, measured as a decrease in the Chao and Shannon indices [13].

### 3.2. Altered GM Composition

While healthy GM is dominated by anaerobic species, SLE is characterized by a decrease in the two primary anaerobic phyla, Firmicutes and Bacteroidetes, and an increase in Proteobacteria aerobic species, which may be more competitive in a host’s inflammatory environment [14]. The main alterations of GM associated with SLE are outlined in Table 1, whereas bacterial species that have been associated with LN are outlined in Table 2.

A reduced ratio between Firmicutes and Bacteroidetes (F/B) appears to be a hallmark of LN, although it has also been observed in other systemic autoimmune disorders [15]. This alteration has been associated with an imbalance in T regulatory cells (Treg) and T-17 lymphocytes and the dysfunction of innate lymphoid cells C3 (ILC3), key contributors to barrier immunity affecting the levels of interferon-γ (IFN-γ). The reduced F/B ratio is probably due to a relative abundance in the genus *Bacteroides*, which belongs to the phylum Bacteroidetes. *Bacteroides* are responsible for inflammation by secreting lipopolysaccharide (LPS) and toxic proteolytic peptides [16]. Interestingly, *Bacteroides thetaiotaomicron* contains an epitope protein homolog that is similar to the human Ro60 autoantigen and was found to elicit the production of autoantibodies and renal deposition of immunocomplexes (IC) through molecular mimicry (Table 2). This mechanism is shared by several species that have the potential to trigger autoimmunity, as analyzed in Section 4.2.

On the other hand, Firmicutes are markedly reduced in SLE and show an inverse correlation with SLE Disease Activity Index (SLEDAI) scores. They produce short-chain fatty acids (SCFAs), such as butyric acid and propionic acid, which can inhibit B cell activity by promoting the proliferation of extrathymic Tregs, suppress the expression of LPS-induced inflammatory cytokines (IL-6, IL-12), and preserve the integrity of the intestinal epithelial barrier function in lupus-prone animals [8]. The role of SCFAs will be further analyzed in Section 4.5.1.

Proteobacteria phylum also appears to be abnormally represented in SLE and is more abundant than in healthy controls, especially in patients with LN. Interestingly, elevated levels of Proteobacteria have also been observed in inflammatory bowel diseases (IBD) and in other nephropathies, such as diabetic kidney disease and membranous nephropathy. This phylum encompasses many Gram-negative pathogenic bacteria, with outer membranes mainly characterized by 44 lipopolysaccharides: these include *E. coli*, which can release LPS and promote the production of IL-6, and *Enterobacteriaceae*, which can stimulate T cell function. *Salmonella* spp., *Shigella* spp., *Vibrio* spp., and *Helicobacter pylori* are also part of this phylum [17]. A recent study on 114 SLE patients has shown a remarkable increase in Proteobacteria/Bacteroidetes ratio as compared to healthy controls and identified *Escherichia* as the most expanded genus. Associations between multiple epitopes from *Escherichia coli* and both disease activity and the presence of LN were found. Furthermore, gavage with fecal *Escherichia* upregulated SLE serum traits and worsened LN in MRL/lpr mice [18] (Table 2).

Actinobacteria, such as *Bifidobacterium* spp., exert beneficial effects on gut health, and an imbalance between Actinobacteria and Proteobacteria can contribute to gut inflammation [17].

At the genus level, a higher abundance of *Streptococcus* genus positively correlated with immunological activity and an enhanced autoimmune response triggered by streptococcal infection may play a role in renal damage, as confirmed by its extreme enrichment in LN [19] (Table 2). Of note, the polysaccharide of *Streptococcus pneumonia* shares the same epitope as the pentapeptide found in the anti-dsDNA antibody. The synergy between the *Streptococcus* and *Veillonella* genus, which is also expanded in SLE [20], results in increased production of several pro-inflammatory cytokines (IL6, IL10, and IL8).

At the species level, a higher abundance of *Ruminocococcus gnavus* (RG), *Ruminococcus torques*, and *Lactobacillus reiteri* has been reported [29], and all of them have been associated with renal involvement (Table 2).

A recent meta-analysis on 92 studies of patients with rheumatoid arthritis, Sjogren’s syndrome, and SLE shared a common pattern of depletion of anti-inflammatory, butyrate-producing microbes (i.e., *Faecalibacterium*) and the enrichment in pro-inflammatory ones (i.e., *Streptococcus*), suggesting the presence of shared mechanisms linking dysbiosis to autoimmunity [13].

### 3.3. Abnormal Activation of Metabolic Pathways

An altered GM composition has a large impact because it results in the activation of several metabolic pathways in SLE [30], such as the sulfur metabolism pathway [23], glycan degradation and mitochondrial oxidative stress [15].

Immunometabolism is currently the focus of intense research and studies on activation of the mechanistic target of rapamycin, calcium signaling, glucose utilization and tryptophan degradation are providing the basis for potential therapeutic targets [31].

For example, a recent analysis based on a metabolomic screening identified an increase in fecal kynurenine, a common tryptophan metabolite, in a mouse model of SLE and found that high dietary tryptophan worsened the disease, whereas dietary restriction had a beneficial effect, as analyzed in Section 4.5.1 [32].

### 3.4. Instability of GM over Time

A longitudinal study has revealed that the composition of GM in patients with SLE is unstable over time, probably lacking resilience to environmental stressors such as infections, food additives and drugs [10]. This would make it prone to dysbiosis and consequently predispose to LGS and systemic inflammation. On the contrary, a healthy GM maintains a dynamic balance over time, with less intense fluctuations, and this is crucial to support mucosal integrity.

Some longitudinal studies also demonstrated that in more than 40% of a small cohort flares of LN occurred in coincidence with blooms of RG, which were 20–90 fold greater than in healthy controls. These studies suggest that GM dysbiosis is characterized by the presence of unstable gut communities, which may quickly expand and cause breaches of immune tolerance [22].

## 4. Pathogenetic Mechanisms Linking Dysbiosis, SLE, and LN

**Figure 2:**
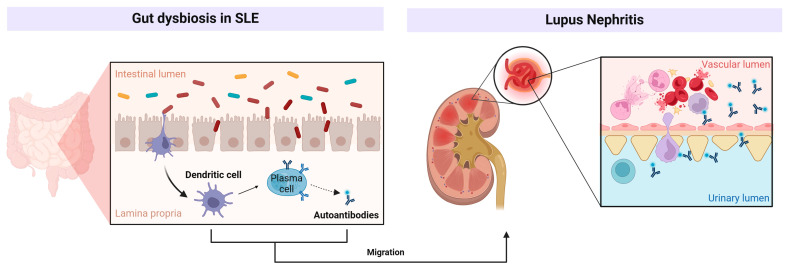
Mechanisms linking dysbiosis and LN (created in BioRender. Merlotti, G. (2025). https://BioRender.com/y27l103 (accessed on 12 February 2025)).

Complex pathogenetic mechanisms mediate the link between dysbiosis on the development of SLE and LN (Figure 2).

### 4.1. Leaky Gut Syndrome (LGS)

LGS is an important predisposing factor to autoimmune disorders. An intestinal barrier deficiency is present in SLE, and expression of the gut-tight junction protein Claudin-1 (cldn1) and Zonula Occludens-1 (ZO-1) appears to be downregulated in LN model mice compared with controls [33]. Some bacterial species, such as Segmented Filamentous Bacteria (SFB), may decrease the expression of cldn1 and ZO-1, while administration of *Lactobacillus* treatment can upregulate it [27,34].

Activation of Toll-like receptor (TLR) 7 and TLR8 appears to mediate breach in the gut barrier in a spontaneous mouse model of SLE but not in non-autoimmune mice, suggesting synergy between autoimmunity susceptibility genes and TLR activation.

An altered gut permeability predisposes to translocations of whole bacteria or gut-derived pathogen-associated molecular patterns (PAMPs) such as LPS or β-glucan from the intestinal lumen into the blood, likely playing a pathogenetic role in SLE [3]. Interestingly, even first-degree relatives of SLE patients are characterized by increased LPS blood levels and a reduced circulating microbiome diversity, which correlate with the presence of pathogenic autoantibodies [35].

The translocation of whole microorganisms has also been demonstrated. For example, the transfer of *Enterococcus gallinarum* from the bowel to draining lymph nodes, the liver and the spleen has been demonstrated in murine models, and the same agent has also been identified in liver biopsy of SLE patients [36]. Interestingly, this human gut commensal bacterium can induce the production of anti-dsDNA autoantibodies [37].

Another key player in this setting is RG, which is expanded in patients with LN and can translocate to mesenteric lymph nodes. Lupus-derived strains of RG can induce higher intestinal permeability, especially in female mice, suggesting a sex-dependent effect. This process was associated with higher serum levels of zonulin, a regulator of gut barrier tight junctions, and completely reversed by an antagonist of this molecule. Interestingly, RG-induced increased gut permeability correlated with both serum IgG antibodies against RG cell-wall lipoglycan and anti-native DNA autoantibodies [38] (Table 2). This mechanism of molecular mimicry will be further analyzed in Section 5.2.

Potential biomarkers of altered intestinal permeability and microbial exposure characterizing LGS have been investigated in SLE with initial interesting results. These molecules are involved in innate immunity with different roles and are generally referred to as “antimicrobial response factors” (ARF). They include a soluble cluster of differentiation 14 (sCD14), lipopolysaccharide-binding protein (LBP), lysozyme, galectin-3, fatty acid binding protein 2 (FABP2), and their circulating levels increase when the integrity of intestinal epithelium is disrupted [39]. Among them, sCD14 is higher in SLE patients than in healthy controls and is associated with the presence of LN and immunological activity [40]. LPB, which participates in inflammatory response triggered by recognition of LPS [41], appears to correlate with lactulose/mannitol ratio, an established biomarker of altered intestinal permeability [42]. Intestinal FABP2, a lipid chaperone that can traffic lipids from the intestinal lumen to enterocytes [43], may be involved in the gut-to-brain propagation of α-synuclein, potentially contributing to the pathogenesis of α-synucleinopathies such as dementia with Lewy bodies and Parkinson’s disease [43]. Thus, these molecules may represent not only biomarkers reflecting LGS but also mediators of immunological crosstalk between the gut and different organs [44].

The presence of LGS has a significant impact on innate and adaptive immunity, predisposing to autoimmunity through a wide range of effects [8], including increased LPS levels and enhanced B-cell activation [34]. After gut colonization of NZM2410 mice with Segmented Filamentous Bacteria (SFB), increased permeability was associated with IC deposition at both glomerular and tubular levels and the development of LN, with an accelerated disease compared to that of the same mice model without SFB. Renal infiltration of M2-like macrophages was detected and associated with higher tissue expression of MCP-1 and CXCL1 (Table 2). An increase in Th17 and ILC3 within gut lamina propria and higher levels of IL17A were also observed [27]. This is a prototypic example of a symbiont species that directly contributes to autoimmune pathogenesis in a susceptible host and is, therefore, referred to as a pathobiont [45].

### 4.2. Molecular Mimicry, Epitope Spreading and Bystander Activation

Molecular mimicry is a key mechanism linking gut dysbiosis to autoimmunity. Several studies have suggested that both bacterial antigens and their metabolites can trigger the production of autoantibodies in SLE patients. For example, bacterial lipopolysaccharides, lipoglycans and teichoic acids can activate the cells of innate immunity by binding to TLRs and other pattern recognition receptors [46].

Furthermore, several gut bacteria harbor epitopes resembling host proteins. These are capable of activating T and B cells, triggering an abnormal production of autoantibodies. For instance, the RNA-binding protein Ro60, a common target of autoantibody responses in patients with SLE and Sjogren’s syndrome, shares sequences akin to human Ro60 epitopes and is found in gut microorganisms such as *Bacteroides thetaiotaomicron* [21]. Anti-Ro60 antibodies can induce the generation of autoantibodies against Ro52, Smith, or U1RNP, progressively expanding the spectrum through epitope spreading [47]. Of interest, the depletion of Ro60 ortholog-expressing bacteria determined a reduction in Ro60-specific T cells. Moreover, *Bacteroides thetaiotaomicron* contains an epitope protein homolog that is similar to the Ro60 autoantigen and can induce autoantibodies and cause IC deposition in a mouse model of LN [21] (Table 2).

Several studies, including cohorts from China, Sweden, and the USA, indicate that an obligate anaerobic commensal RG of the Lachnospiraceae family may play a role in creating a LGS in active SLE, as already dealt with in Section 5.1 [38]. A five-fold increase in its abundance correlated with disease activity, especially if LN was present. This subset of patients displayed the highest levels of serum IgG antibodies to a cell wall lipoglycan of RG, which represents a strain-specific antigen [22]. A Chinese study including 117 untreated SLE patients (including 22 with LN) demonstrated that expansions of RG were related to flares of LN [23]. Whole genome sequence analysis of RG strains isolated during flares identified 34 genes that are not present in healthy controls and may favor adaptation within an inflammatory milieu. In addition, these strains shared expression of a cell membrane-associated lipoglycan with highly immunogenic antigenic determinants recognized by serum IgG2 antibodies. Anti-RG antibody titer actually increased in coincidence with RG blooms and lupus flares and correlated directly with SLEDAI and anti-DNA levels and inversely with C3 and C4 [10]. Furthermore, two related cell wall-anchored proteins of RG, immunoglobulin-binding proteins A and B, can exert a B cell superantigen-type effect [48].

Several Staphylococcal antigens are also capable of generating nephritogenic immune complexes, and DNABII proteins within the biofilm of *S. aureus* can trigger the production of anti-HU1 autoantibodies, which cross-react with an antigen expressed on renal cells [25,49] (Table 2).

Increased levels of some Heat-Shock Proteins (HSP) also appear to trigger the production of anti-HSP autoantibodies, which correlate with SLE onset and progression. Molecular mimicry by shared epitopes between human HSPs and those of gut commensal bacteria is a plausible mechanism [50].

Two microbial peptides, YLYDGRIFI and DGQFCM, can induce autoimmunity via molecular mimicry. The peptide YLYDGRIFI, derived from *Odoribacter splanchnicus*, resembles the Sm autoepitope and can elevate IFNγ levels and IL-17A release by peripheral blood mononuclear cells in some anti-Sm-positive SLE patients. The peptide DGQFCM, isolated from *Akkermansia muciniphila*, is similar to the Fas autoepitope sequence and is the target of IgG released by memory B lymphocytes from SLE patients [23]. The fecal abundance of *A. muciniphila*, which is frequently observed in SLE, appears to strongly correlate with blood IgM and IgA levels and all markers of inflammation [23].

Also, microbiota-derived mimotopes can represent a persistent trigger in human autoimmunity. In an antiphospholipid syndrome (APS) model, cross-reactivity between non-orthologous mimotopes expressed by a common human gut commensal, *Roseburia intestinalis* (RT), and T/B cell autoepitopes β2-glycoprotein I (β2GPI) was shown. IgG titers of anti-RT mimotope were elevated in APS patients and correlated with anti-β2GPI IgG autoantibodies and also thrombotic events [51].

Finally, NETosis can indirectly promote molecular mimicry. The interaction between gut bacteria and neutrophils can trigger this process, which is characterized by the release of highly immunogenic material, including commensal antigens, which are orthologs of self-antigens such as Ro60 [52].

Other potential mechanisms that can predispose to autoimmunity are represented by bystander activation, superantigens, persistent viral infections [53], defective apoptosis and impaired IC clearance, and epigenetic alterations [54].

### 4.3. Immune Dysregulation

Dysbiosis is linked to a wide range of significant alterations in both innate and adaptive immunity in SLE [8,55].

#### 4.3.1. Innate Immunity

Dysbiosis can determine an imbalance of the innate immune system function of several immune cell types. Under normal conditions, GM regulates the physiological functions of gut-associated lymphoid tissue (GALT), such as ILC3 proliferation, the production of IL-22 and the release of protective proteins of the intestinal mucosal barrier [8]. Microbiota-derived SCFAs mediate most of these actions and modulate the response of GALT through epigenetic mechanisms [56]. GM can also modulate NK cell cytotoxicity and myeloid cell hematopoiesis and migration within the gut, shaping the gut immune microenvironment. All these innate immunity cell functions are altered in SLE, contributing to the activation of TLRs and inflammasome [56,57].

#### 4.3.2. Adaptive Immunity

GM plays a key role in the differentiation of naïve CD4^+^ T cells into Th17 cells and Treg cells, which are essential in defending the gut from infections on the one hand while preserving tolerance on the other. An increase in Th17/Treg ratio can be found in SLE due to dysbiosis, and the role of some pathobionts, such as SFB, in stimulating Th17 has been shown [58].

In general, a reduced F/B ratio also correlates with Th17 differentiation and lymphocyte activation and a reduced biosynthesis of SCFAs, whereas it negatively correlates with SLEDAI [59]. On the contrary, the administration of bacterial strains of Firmicutes phylum reduced IL-17 and IFNγ production and prevented T-lymphocyte overactivation [14]. Butyric acid and propionic acid, SCFAs mainly produced by phylum Firmicutes, inhibit B cell activation and production of autoantibodies by promoting the differentiation of extrathymic Treg cells and suppressing the expression of LPS-induced inflammatory cytokines in animal models [60] (Table 3).

The link between a high-fiber diet and high SCFA levels and beneficial immune modulatory effects in mice has been confirmed by a recent review, which indicates that a low-fiber diet worsens the deposition of ICs in kidney glomeruli [66].

The colonization of *Lactobacillus* reversed LGS in MRL/lpr mice and reduced inflammation both in the gut (decreased IL 6 and increased IL 10 production) and in the circulation (decreased nephritogenic IgG2a). This treatment succeeded in skewing the Th17/Treg balance towards a Treg phenotype in the kidney, but only in female and castrated male mice, suggesting a hormone-dependent beneficial effect [34].

Also, *Lactobacillus fermentum* appears to be effective in Th17 infiltration in kidney tissue and to improve renal parameters in mouse models of hypertensive SLE [61].

Progress in understanding the relationship between microbiota and adaptive immunity is paving the way for novel treatments with probiotics, which will be analyzed in Section 5.3.2.

### 4.4. The Role of Sex Hormones

Sexual hormones can interact with GM in healthy women. Accumulating evidence indicates that prolactin and especially estrogen can shift GM to a pro-inflammatory state, potentially overriding self-tolerance, whereas progesterone and testosterone exert opposite actions [67]. A direct correlation was found between high estradiol levels and a decrease in the F/B ratio due to a decrease in Firmicutes and an increase in Bacteroidetes [68].

Furthermore, significant differences exist in GM composition in young, female lupus-prone mice resembling women at childbearing age: marked depletion of Lactobacilli and an increase in Lachnospiraceae were found compared to age-matched healthy controls. In addition, GM in this lupus-prone model was different between sexes, and the overrepresentation of Lachnospiraceae in females was associated with an earlier onset of more severe disease [55]. Consistently, Edwards et al. detected an increase in Lachnospiraceae and a strong progression of SLE manifestations in female MRL/lpr mice that were fed diets enriched with phytoestrogens [69]. Pregnancy and lactation also have a profound impact on GM composition and may favor a pro-inflammatory profile [70]. Furthermore, they also modify the response to therapeutic modulation of GM; for example, vancomycin was effective in expanding *Lactobacillus animalis* in a mouse model in which it was administered outside of pregnancy, whereas it was ineffective in the same model when administered post-partum, possibly due to differential expression of IL10 and INFy between the two conditions [71].

### 4.5. The Role of Environment

Several environmental factors can modulate GM and make it more prone to stimulate autoimmunity [72]. Diet, age, gender, race, and BMI may affect the composition of intestinal flora [73,74].

#### 4.5.1. Diet

Daily diet can probably modulate immune function in SLE [66] and part of this effect is mediated by changes in GM composition and diversity [75].

A low-fiber diet can promote IC deposition in kidney glomeruli in C57BL/6J mice [66] and an increase in white adipose tissue mass and low-grade inflammation in other models [76]. On the contrary, a resistant starch (RS)-rich diet generating SCFAs has been shown to suppress *Lactobacillus reuteri*, known for exacerbating TLR7-dependent SLE activity, thus inhibiting IFN pathways and reducing SLE symptoms in transgenic mice reproducing TLR7-pDC-IFN axis [77].

**Figure 3:**
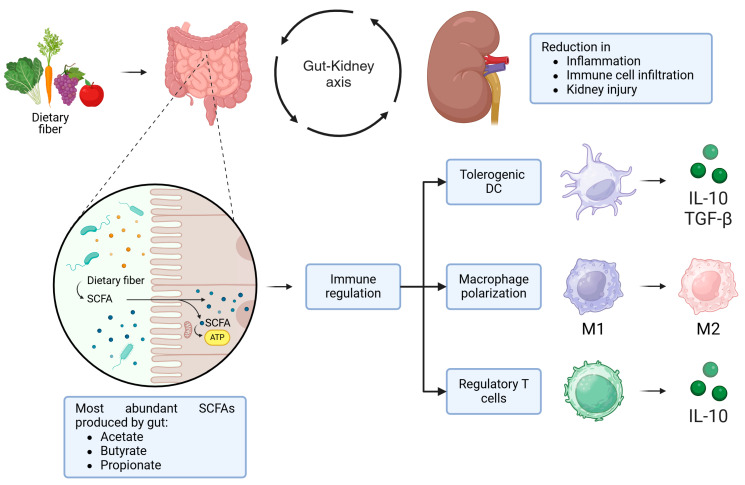
The immunomodulatory role of short-chain fatty acids in SLE and LN (created in BioRender. Merlotti, G. (2025) https://BioRender.com/w96z110 (accessed on 12 February 2025)).

Many beneficial immunomodulatory and anti-inflammatory effects associated with diet fibers and non-digestible carbohydrates are mediated by SCFAs, such as acetate, butyrate and propionate (Figure 3).

All SCFAs are generated by microbial fermentation of dietary fiber in the gut and modulate many cells of innate immunity (ILCs, DCs, macrophages) and adaptive one (T and B cells) through G-protein coupled receptors and epigenetic regulations of gene expression by histone deacetylase (HDAC) inhibition [78]. The latter mechanism is involved in the expansion of peripheral and colonic Treg [79] and the inhibition of B cells’ somatic hypermutation and their differentiation into plasma cells [80].

While Bacteroides and Negativicutes groups produce propionate, butyrate is mainly released by Firmicutes phylum [81]. Butyrate is essential to maintain gut homeostasis as it is involved in colonocyte energetic metabolism and intestinal barrier integrity and exerts anti-inflammatory actions [82].

A high tryptophan diet exerted a pro-inflammatory effect and was associated with elevated levels of *Lactobacillus* spp. and *Bacteroides dorei* and the expansion of Th17 in triple congenic lupus-prone mice; conversely, a low tryptophan diet improved Treg function [32]. Tryptophan, which is characterized by abnormal catabolism in SLE and other inflammatory states [83], exerts immunoregulatory actions by producing ligands of aryl hydrocarbon receptor, a cytoplasmic receptor and transcription factor which plays an important role in mediating the impact of environmental factors on SLE [84]. Interestingly, dietary fermentable fibers inhibit the production of indole, a tryptophan metabolite, but promote the generation of other tryptophan metabolites associated with health benefits [85].

#### 4.5.2. Drugs

Drugs play an important role among environmental factors that modulate GM [49], and SLE patients have chronic exposure to glucocorticoid (GC), hydroxychloroquine (HCQ) and immunosuppressive drugs. GC has beneficial effects on GM, increasing the F/B ratio by expanding Firmicutes and consequently promoting SCFA production and mucosal integrity [86]. On the contrary, HCQ exerts detrimental effects by increasing the F/B ratio [87].

The effect of antibiotic therapy has a profound impact on GM. Antibiotics often eradicate Bacteroidetes, Firmicutes and Actinobacteria taxa, creating a niche for opportunistic bacteria associated with gastrointestinal disease and autoimmunity, such as Proteobacteria [88]. On the other hand, targeted antibiotic treatment has been employed to correct some aspects of dysbiosis, as discussed in Section 5.2.

Gastroprotection with proton-pump inhibitors, which is routinely employed in SLE patients, can also significantly alter GM, affecting around 20% of bacterial taxa and decreasing diversity; a significant increase in bacteria of genera *Enterococcus*, *Streptococcus*, *Staphylococcus* and in the potentially pathogenic species of *Escherichia coli* were also observed [89].

## 5. Therapeutic Manipulation of Gut Microbiota to Treat LN

**Figure 4:**
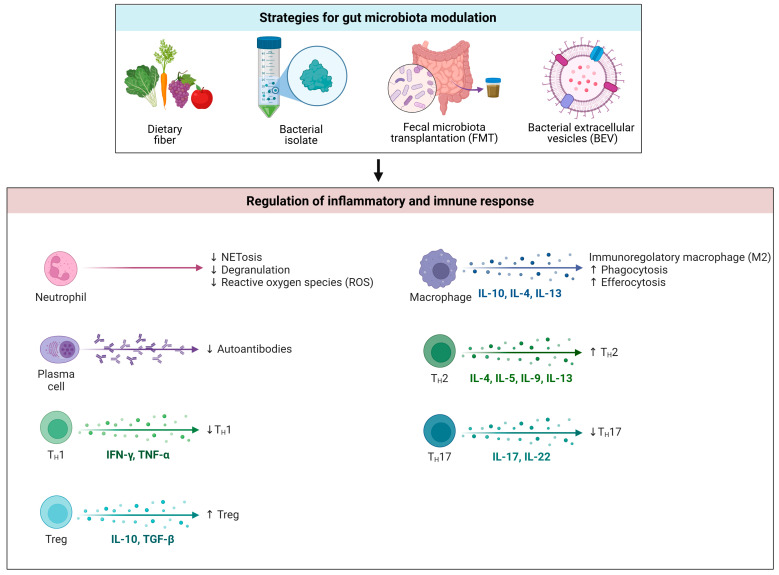
Approaches to GM modulation in SLE (created in BioRender. Merlotti, G. (2025) https://BioRender.com/y27g141 (accessed on 12 February 2025)).

Therapeutic manipulation of GM is a promising option to reduce inflammation in SLE and potentially blunt LN and other disease manifestations [90]. Reshaping of GM can be performed through several approaches, from diet to the administration of antibiotics or prebiotics, probiotics, symbiotics, and fecal transplantation [91]. Novel tools such as the administration of bacterial extracellular vesicles (BEVs) are also promising in the field of autoimmunity. We will analyze these treatments, also including some studies performed in animal models of rheumatoid arthritis (RA) and IBD, as these share LGS with SLE (Figure 4).

### 5.1. Diet Intervention

Diet interventions have been investigated with the aim of correcting GM imbalances that characterize SLE [19] (Section 4). A reduced F/B ratio is associated with impaired butyrate production in SLE patients, and butyrate administration for 8 weeks has been shown to increase the F/B ratio and blunt renal damage, reducing mesangial cell proliferation, matrix expansion and interstitial inflammation in lupus-prone mice [82,92]. It also directly promotes efferocytosis in mouse and human macrophages, favoring clearance of apoptotic cells [93], and can reduce levels of different autoantibodies (anti-dsDNA, anti-Sm) through epigenetic reprogramming of B lymphocytes [80]. Furthermore, both direct supplementation of butyrate and of fibers increasing butyrate-producing bacteria exert multiple cardiovascular protective effects by reducing blood pressure, left ventricular hypertrophy, oxidative stress, Th17 intra/aortic infiltration and endothelial dysfunction [94]. Overall, targeting microbiota with dietary appears to be a promising novel approach to reduce cardiovascular risk [95]. However, the role of RS-rich diet and SCFAs requires further study [77], especially due to the multiple, complex actions of SCFAs in SLE [56] (Figure 3).

A low-tryptophan intake has also been proposed to reduce disease activity [32].

A systematic review has recently concluded that a low-calorie, low-protein diet with elevated contents of RS, fibers, polyunsaturated fatty acids, vitamins, minerals and polyphenols could help reduce inflammation [96] and prevent cardiovascular complications in this high-risk population [95].

### 5.2. Antibiotics

Oral mixed antibiotics given during active disease blunted lupus-like symptoms in MRL lpr mice, improving cytokine profile (increase in circulating IL/10 and decrease in IL/17 levels) and GM balance. The latter effect consisted of a reduction in Lachnospiraceae and an expansion of *Lactobacillus* spp. Moreover, improved renal histology was shown. These beneficial effects could be reproduced by a single infusion of vancomycin, which has been shown to prevent LPS translocation into circulation and protect intestinal epithelium [97].

On the other hand, broad-spectrum antibiotics and also vancomycin have been shown to disrupt efferocytosis in murine models, an essential process that prevents autoimmunity [93]. As discussed in Section 4.5.2 the use of antibiotics generally alters GM, reducing physiological populations such as Firmicutes and creating an inflammatory environment with a rise of pathobionts [88]. Further studies are needed to define the potential therapeutic role of this approach in modulating GM in SLE.

### 5.3. Prebiotics, Probiotics, and Synbiotics

#### 5.3.1. Prebiotics

Prebiotics are defined as “a substrate that is selectively utilized by host microorganisms conferring a health benefit”. Thus, the concept includes a substance, a physiologically beneficial effect, and a microbiota-mediated mechanism.

In a recent study on the Brazilian population, the content of fiber and calcium intakes was below recommended and most patients had sedentary behavior and low functional and aerobic capacity compared to the general population [98].

Curcumin, a polyphenolic compound isolated from turmeric rhizomes [99], and resveratrol [100] both alleviate inflammation in SLE patients, reducing anti-ds DNA and IL-6 levels. Other dietary polyphenols, such as dihydrochalcones and flavanones, modulated GM and improved lupus disease activity [75].

Interestingly, an association between prebiotics and specific microbial genera has been reported in SLE. For example, an association has been shown between flavone intake and *Blautia*, flavanones and *Lactobacillus*, dihydrochalcones and *Bifidobacterium* [101]. Further studies are needed to investigate the impact of dietary prebiotics on SLE.

#### 5.3.2. Probiotics

Probiotics are live microorganisms that confer health benefits, primarily on the gastrointestinal tract. Probiotics mostly belong to Gram-positive bacteria such as *Lactobacillus, Bifidobacterium, Streptococcus, Saccharomyces, Bacillus,* and *Enterococcus* [102]. They have been employed in several SLE animal models in which they have shown interesting tolerogenic and nephroprotective effects [64]. A recent review identified 12 studies in which probiotic strains were administered for a period of 8–47 weeks. These included *L. fermentum* CECT5716, *L. casei* B255, *L. reuteri* DSM 17509, *L. plantarum* LP299v and *L. acidophilus* and had a significant impact on four key aspects: (a) reduction of pro-inflammatory cytokines (TNF-α, IL-12, IL-6, IL-1β, IL-17, and IFN-γ) and increase in anti-inflammatory IL-10; (b) expansion of Treg cells and shift in Th17/Treg ratio; (c) delayed production of autoantibodies; (d) protection of barrier integrity [103]. The role of probiotics in LN is summarized in Table 3.

*Lactobacillus fermentum* CECT5716 (LC40) administration has determined a reduction of circulating anti-dsDNA production and LPS and inhibited IC deposition in the kidney. This resulted in improved renal function and decreased albuminuria, suggesting that it could have a therapeutic effect on LN in the NZBWF1 mouse model [61].

In another study, a mixture of five *Lactobacillus* strains (*Lactobacillus oris, Lactobacillus rhamnosus, Lactobacillus reuteri, Lactobacillus johnsonii*, and *Lactobacillus gasseri*) decreased circulating IgG2a, an immune deposit observed in the kidney of MRL/lpr mice. Lactobacillus treatment also skewed the Treg-Th17 balance towards a Treg phenotype within kidney tissue. These beneficial effects, which were largely due to *Lactobacillus reuteri*, were present in female and castrated male mice but not in intact males, suggesting that GM controls LN interacting with sex hormones. Furthermore, Lactobacillus strains were administered before full-blown disease, indicating a possible prevention of the development of LN [34]. This beneficial effect can be explained by its regulatory effects on intestinal host epithelial cells, immune cells, cytokines, TLRs, tryptophan metabolism, antioxidant enzymes, and anti-inflammatory effects, leading to the alleviation of disease symptoms [104].

Interestingly, *Lactobacillus acidophilus*, which can induce Treg, enhanced the efficacy of Tacrolimus by elevating IL-10, reducing IL-17 and modulating the Th17/Treg ratio in a mouse model. This suggests a possible synergy with calcineurin inhibitor-mediated immunosuppressive effects on T-cell function [62].

*Lactobacillus rhamnosus* and *Lactobacillus delbrueckii* can blunt the activity of miR-155 and miR-181 in peripheral blood mononuclear cells of SLE patients. Reduced expression of these miRs, which both correlate with disease severity, has been associated reduction of anti-dsDNA titers and alleviated disease symptoms [63].

Furthermore, both species can promote an anti-inflammatory profile of macrophage-derived monocytes isolated from newly diagnosed patients with SLE, potentially treating an imbalance of M1/M2 macrophages [64].

Other probiotics which have been employed in pre-clinical models of LN are outlined in Table 3. In a recent meta-analysis, Zeng L et al. analyzed available RCTs on GM-based therapy in SLE and concluded that, in general, probiotics have proven effective in reducing SLEDAI and total IgG levels and in increasing C3 complement fraction, with a good safety profile [105].

However, despite growing evidence in animal models, there is a paucity of studies in humans. In a cross-sectional study carried out in Taiwan use of probiotics was associated with a significantly lower risk of photosensitivity and renal involvement [106]. Another study suggests that they may reduce cardiovascular risk in patients with SLE [107].

#### 5.3.3. Synbiotics

Synbiotics are associations of prebiotics and probiotics, which show an increased effectiveness compared to probiotics and prebiotics alone.

Widhani et al. administered a synbiotic supplement (including *Lactobacillus helveticus, Bifidobacterium infantis, Bifidibacterium bifidum* and fructooligosaccharides) for 2 months within a randomized, double-blind, placebo-controlled trial on 46 adult Indonesian patients with SLE and gastrointestinal symptoms. This treatment was effective in increasing butyrate-producing bacteria and metabolism, blunting systemic inflammation (decreased hs-CRP and IL6 levels) and immunological activity (reduction in SLEDAI-2k), and increasing the F/B ratio [108].

Consistently, Zhu et al. showed that administration of synbiotics to patients with newly diagnosed LN decreased the abundance of Bacteroides and Proteobacteria while increasing that of Firmicutes and Actinobacteria, indicating that treatment affected both pathogenic and beneficial phyla, correcting main alterations consistently reported in SLE (Table 1); furthermore, at the genus level, it affected *Bacteroides*, *Parabacteroides*, *Faecalibacterium* and *Prevotella* [109]. A reduction in the abundance of *Prevotella* may be especially important as its expression was upregulated before treatment and it has been associated with the development of LN [13]. A metabolomic analysis in the same study also demonstrated that amino acids biosynthesis, aminoacyl-tRNA biosynthesis, purine metabolism and other metabolic pathways may be altered in patients with LN and could be effectively corrected after the addition of synbiotics [109].

### 5.4. Fecal Microbiota Transplant (FMT)

Transferring microbiota from lupus-prone mice into healthy ones can induce the disease and germ-free mice receiving fecal matter from SLE mice developed increased levels of anti-dsDNA antibodies and a lupus-like phenotype. In a recent study, fecal microbiota from MRL/lpr mice exacerbated pristane-induced lupus, affecting the immune cell profile (especially plasma cells) and reshaping the recipient’s GM. An abundance in Prevotella taxa was associated with the activation of several metabolic pathways and the development of proteinuria in the pristane-induced lupus mice, suggesting the impact of FMT from a pathological donor in conditioning SLE phenotype in recipients [28].

The concept of using FMT from a healthy donor to reconstitute physiological gut-immunity homeostasis has a strong biological rationale, as gut dysbiosis is central to SLE pathogenesis. In 2022, the first single-arm pilot clinical study (EXPLORER trial) assessed the effectiveness of oral encapsulated fecal microbiome (weekly administration for 3 consecutive weeks, in addition to standard treatment) from healthy donors to 20 patients with active SLE (SLEDAI ≥ 6). After 3 months, this approach achieved a SLE Responder Index-4 response rate of 42.12%, along with significant reductions in the SLEDAI and anti-dsDNA antibody titer. Furthermore, recipients’ GM was enriched in SCFAs-producing bacterial taxa, with a positive effect on circulating levels of IL-6 and CD4^+^ memory/naïve ratio. No safety issues were reported [110,111].

Another study explored the mechanisms underlying FMT effectiveness and found that the serum level of S-adenosylmethionine, a methylation group donor, was upregulated after FMT, resulting in an increase in genome-wide DNA methylation levels in responders. Interestingly, methylation levels in critical regions, including promoter of IFN-γ, increased after FMT [112]. Promoting recovery from a state of abnormal hypomethylation is a promising rationale, as IFN-regulated genes are hypomethylated in naïve CD4^+^ T cells, CD19^+^ B lymphocytes and CD14^+^ monocytes [113].

### 5.5. Bacterial Isolates and Bacterial Extracellular Vesicles (BEVs)

An alternative, more selective approach compared to FMT has also recently been attempted in order to minimize the risk of introducing pathogenic bacteria into the gut, which consists of the administration of specific bacterial strains from healthy stool [114]. Modulation of GM with *Akkermansia muciniphila*, *Bifidobacterium* spp. and *Faecalibacterium prausnitzii* was able to restore the gut barrier in IBD [115], and the consumption *of Bifidobacterium bifidum* isolated from fecal samples obtained from a healthy control (4 weeks) resulted in a significant increase in the Ruminococcaceae and Rikenellaceae bacterial families and butyrate fecal levels and a decrease in the Prevotellaceae one in healthy recipients studied within a RCT [116]. Despite limited data in humans, these results suggest a potential application in SLE patients [117].

Another potential tool with a similar rationale is that of bacterial extracellular vesicles (BEVs), which can modulate microbial ecosystems, contributing to pathogenicity and influencing the host’s immune response [118]. Extracellular vesicles (EVs) are a heterogeneous group of lipid-membrane-bound vesicles secreted by almost all cell types, which play an important role in intercellular communication; their complex bioactive cargo includes microRNA and other genetic material, through which they can genetically reprogram target cells [119]. EVs released from food [120] and BEV are likely to play an important role in gut-kidney crosstalk and may modulate chronic kidney inflammation [121]. *Akkermansia muciniphila*-derived EVs were shown to inhibit the production of IL-6 from colon epithelial cells and reduce mucosal inflammation in an IBD mouse model [122], and Lactobacillus-derived EVs have been proposed as a therapy for IBD and other AIDs [123].

## 6. Beyond Bacteria: The Role of Viruses and Yeasts in SLE and LN

Beyond bacterial components of GM, the virome—including eukaryotic viruses, bacteriophages, prophages, and endogenous retroviruses—and the mycobiome—which comprises fungal communities such as yeast—play an increasingly recognized role in the modulation of immune response and stability of gut homeostasis [124,125]. While fungi account for nearly 0.1% of the total microbes in the gut, viral particles are estimated to be 10^13^ per human individual. However, the majority of sequence data in a typical virome study remain unidentified, highlighting the extent of this unexplored viral ‘dark matter’ [126,127]. Imbalances in these microbial ecosystems can contribute to AIDs through various mechanisms, including chronic inflammation and immune dysregulation [128,129,130,131].

As for virome, an increased level of Iridoviridae, Drexlerviridae, Herelleviridae, and Phycodnaviridae families was observed in the SLE. Enrichment of these taxa in SLE patients suggests that they may play a role in immunosuppressed subjects [132]. Moreover, analysis of phages in a Japanese cohort showed that *Podoviridae* were significantly decreased in the gut of the patients with SLE in parallel with *Faecalibacterium* spp., thus highlighting a symbiotic relationship [133]. Interestingly, viruses that are predicted to infect *Bacteroides*, *Parabacteroides*, and *Ruminococcus* appear to be enriched in the SLE patients; increased abundance of *Bacteroides fragilis* and *Ruminococcus gnavus* and their specific roles in LN (Table 2) suggests that interaction between these bacterial species and related phages may be involved in etiopathogenetic mechanisms of the disease [12,23]. Herpesviridae, particularly Human Herpes Virus (HHV)3, HHV4, and HHV5, represent another viral family associated with SLE. An elevated SLEDAI was observed in patients with lytic HHV4 infections and HHV-5 appears to exacerbate SLE [54,134]. Both HHV4 and HHV5 may trigger an abnormal immune response [124]. In addition, a direct link between SLE gut virome and IFN-α production was observed. This molecule is the main constituent of type I IFNs, and its sustained activation in epithelial cells and primary human immune cells (neutrophils, T cells, and B cells) promotes autoantibody production and organ damage in SLE [135].

Research on fungal communities has advanced much more slowly if compared to that on bacterial or viral ones due to their lower abundance and lack of a comprehensive reference genome database; however, their role has been emerging as another predisposing factor for SLE and AIDs [136]. An increase in Candida species (particularly *C. albicans* and *C. glabrata*) and a decrease in *Rhizopus* and *Malassezia* were observed in gut mycobiome of LN patients, providing evidence of host-fungal interactions in SLE and suggesting potential application as fungal biomarkers [137]. As for *C. albicans*, this yeast plays a key role in enterocyte inflammation by pseudohyphae and biofilm formation, supporting the growth of certain bacteria [138,139]. Moreover, Candida-derived mannans can engage TLRs and other innate immunity receptors, triggering the production of pro-inflammatory cytokines [140].

An interesting aspect is the role of SCFAs in promoting antifungal activity, as sodium butyrate can inhibit the growth and filamentation of *C. albicans* and enhance the antimicrobial actions of macrophages in response to *C. albicans* sensing [141]. Thus, bacterial dysbiosis could lead to a reduction in SCFA production and an overgrowth of fungal taxa, exacerbating GM alterations within a complex feedback loop between bacteria and fungi [142]. Finally, expanded *Aspergillus* spp. also appears to be associated with inflammation, proteinuria, anti-dsDNA, ANA, and SLEDAI [137,143].

In-depth studies encompassing all microbial kingdoms are necessary to unravel the intricate relationship between microbiome, virome and mycobiome in SLE and LN, as alterations in this network are likely to be crucial in perpetuating immune dysregulation and organ damage.

## 7. Limits and Perspectives

GM and its metabolites are increasingly recognized as major environmental factors capable of triggering and modulating the immunological activity of several AIDs, including SLE.

However, many limits still need to be overcome. Mechanisms linking gut dysbiosis and autoimmunity are only partly known. The classical mechanisms of bacterial translocation, molecular mimicry, epitope spreading and bystander activation provide general pathophysiological background, but further studies are needed to identify potential molecular targets within this process [144] and also explore the potential role of fungi and viruses in pathogenesis [145].

Another area of uncertainty is the influence of GM on the metabolism, drug disposition, action, and toxicity of immunosuppressants, which could have an impact on the efficacy and toxicity of these drugs. Pharmacomicrobiomics is starting to enable us to better understand the interaction between IS drugs and GM and possibly exploit them to improve therapy [146].

Longitudinal studies are needed to follow up changes in the microbiome and immune system over time with repeat sampling, investigating dynamic, mutual interaction between the two systems. Identification of specific bacterial species that expand before or in association with renal flares could pave the way for specific anti-microbial therapy or prophylaxis with pre/probiotics in order to reduce this population to its original weight, restore a physiological balance, and potentially prevent flares. The paradigm of RG is very interesting in this setting [12], but evidence of bacteria associated with LN is limited to a few species so far (Table 2). Microbial network analysis could help broaden our knowledge of all species having nephritogenic properties and their associated antigens and metabolites, which could hopefully provide new therapeutic targets or prognostic tools. Furthermore, targeting bacteria that fuel autoimmunity through intra- and intermolecular epitope spreading could help prevent disease progression from moderate mesangial forms to more aggressive proliferative classes [147].

Microbiome-based therapies can be envisaged as a new form of personalized, neoadjuvant treatment to restore immune tolerance in SLE [146]. They encompass a wide range of interventions, from diet to administration of prebiotics and probiotics up to FMT. Probiotics have been widely employed as part of IBD treatment, and their effectiveness in enhancing tight junctions and reducing LPS production may also be applied to SLE. Schisadrin C, an active compound from the plant *Schisandra chinensis* [148], and probiotics, such as *Bifidobacterium longum* NK219, *Lactococcus lactis* NK209, and *Lactobacillus rhamnosus* NK210, may be considered within this approach [149].

FMT has been recently successfully employed as a rescue treatment in some glomerulonephritis, especially IgA nephropathy, and might, therefore, represent an interesting option to treat resistant and relapsing forms of LN and as an alternative for patients who do not tolerate side effects of traditional immunosuppressive drugs [150]. These initial studies have consistently shown that FMT can reduce proteinuria and stabilize renal function and is well tolerated. However, we still need to clarify many aspects of its therapeutic role, such as dosage regimen and modality of donor screening, and limits due to clinical, biological, and procedural factors, which may influence its efficacy [151]. Some RCTs are underway and may help define new intestinal interventions for SLE patients [111].

## 8. Conclusions

Gut dysbiosis and a LGS have been increasingly recognized as an important environmental factor in SLE pathogenesis. Molecular mimicry and several other mechanisms of immune dysregulation can lead to IFNγ production and alter the balance between T reg and Th17 subsets, triggering disease onset and conditioning immunological activity. Expansion of specific bacterial populations and reduction of others have been associated with LN flares. Levels of bacterial metabolites, such as butyrate and other SCFAs, play an important role in this setting. On the other hand, microbiota-based therapy appears promising and includes diet, prebiotics, probiotics, synbiotics, and also FMT. Harnessing GM and correcting critical alterations, such as the F/B ratio, may help prevent flares and organ damage, including LN. Moreover, understanding the in-depth relationships between microbiome, virome, and mycobiome could offer new opportunities for further targeted therapies. Rebalancing these microbial ecosystems through phage therapy, dietary interventions, probiotics, and FMT has the potential to reduce disease activity, prevent flares and mitigate organ damage.

All these aspects may potentially reduce the need for immunosuppressive drugs or increase their effectiveness and tolerability in the future, modulating a primary and long-underrecognized etiopathogenetic mechanism of disease. Further research into microorganisms–SLE interactions and their immunological implications will pave the way for precision medicine approaches tailored to the unique microbial profiles of individual patients.

## Figures and Tables

**Table 1:** Main alterations of gut microbiota (GM) composition associated with systemic lupus erythematosus (SLE) and potential pathogenetic mechanisms.

Taxonomic Level	Types of Bacteria	Pathogenetic Mechanisms	References
Phylum	Bacteroidetes	It produces short-chain fatty acids (SCFAs) acetate and propionate; however, an increased abundance can determine a reduced Firmicutes/Bacteroidetes (F/B) ratio, which is associated with SLE severity. Taxonomic chain Bacteroidetes*-Bacteroides*-*Bacteroides thetaiotaomicron* is enriched in gut of patients with lupus nephritis (LN).	[15,16]
	Firmicutes	They produce high amounts of butyrate and have a negative correlation with SLE Disease Activity Index (SLEDAI). They promote extrathymic Treg cells and suppress Lipopolysaccharide (LPS)-induced inflammatory cytokines (IL-6, IL-12).	[8,13]
	Proteobacteria	Increased Proteobacteria/Bacteroidetes ratio in SLE as compared to healthy controls; they include many pathogenic bacteria (*Escherichia coli*, *Enterobacteriaceae*), which may have a role in SLE.	[17,18]
	Actinobacteria	Decreased in SLE, they have anti-inflammatory effects.	[17]
Genus	*Streptococcus* spp.	Associated with increased levels of IL-6, IL-10, IL-8, and TNF-α, they enhance autoimmune T and B lymphocyte clones.	[19]
	*Veilonella* spp.	Synergistic effect with *Streptococcus.*	[20]

**Table 2:** Main bacterial species associated with LN and potential pathogenetic mechanisms.

Bacterial Species	Pathogenetic Mechanisms	Setting	References
*Bacteroides thetaiotaomicron*	It contains an epitope protein homolog that is similar to Ro60 autoantigen and induces autoantibodies production and immunocomplex (IC) deposition in LN.	Mice	[13,21]
*Ruminococcus gnavus* (RG)	It can elicit production of IgG towards strain RG2, which correlates with active LN. Cell wall lipoglycans of this strain can induce production of anti-dsDNA through molecular mimicry.	Human	[10,12,22,23]
*Ruminococcus torques*	Enriched in SLE and Sjogren.	Human	[24]
*Staphylococcus aureus*	Associated with anti-HU1 antibodies	Human	[25,26]
Segmented Filamentous Bacteria (SFB)	They determine increased MCP1 and CXCL1 serum and renal levels and kidney infiltrating CD206+ M2-like macrophage infiltration, enhancing glomerular and tubular IC deposition and interstitial inflammation.	Mice	[27]
*Lactobacillus reuteri*	After translocation, it can stimulate activation of plasmacytoid dendritic cells (pDCs) and production of type I Interferon (IFN) in spleen and ileum, enhancing leukocyte recruitment to the kidney.	Mice	[16]
*Streptococcus pneumoniae*	Extremely enriched in LN models, it crossreacts with antDNA through molecular mimicry and synergizes with Veillonella in stimulating production of pro-inflammatory cytokines.	Mice	[19,20]
*Escherichia coli*	Associations between multiple epitopes from *Escherichia coli*, disease activity, and presence of LN. Gavage with fecal *Escherichia* worsened serological profile and LN.	Mice	[18]
*Prevotella* spp.	Expanded in SLE and associated with LN and proteinuria, it decreases after immunosuppressive or probiotic treatment.	Mice	[13,28]

**Table 3:** Immunomodulating role of probiotics in LN.

Probiotic	Mechanism	References
*Lactobacillus fermentum* CECT5716 (LC40)	Decreased circulating anti-dsDNA and LPS and inhibited IC deposition in kidney.	[61]
*Lactobacillus reuteri*	Skewed Treg-Th17 balance; decreased circulating IgG2a and IC deposition in kidney.	[34]
*Lactobacillus acidophilus*	Enhanced efficacy of tacrolimus by elevating IL-10, reducing IL-17, and modulating the Th17/Treg ratio.	[62]
*Lactobacillus rhamnosus* and *Lactobacillus delbrueckii*	Blunted activity of microRNA (miR) 155 and miR-181 and reduction of anti-dsDNA titers; promotion of anti-inflammatory phenotype in macrophages-derived monocytes.	[63,64]
*Bacteroides fragilis* (ATCC 25285)	Improved LN and reduction of anti-dsDNA titers; restored Th17/Treg balance and regulation of CD1d and CD86 expression on B cells.	[65]
*Bifidobacterium bifidum* LMG13195	Increased Foxp3 expression; polarization of naive CD4^+^ T cells towards Treg rather than Th17 cells.	[59]

## Data Availability

The data employed for conducting this review are available upon request to the following e-mail: marco.quaglia@med.unipo.it.

## List of Abbreviations

AID	Autoimmune disorder
APS	Anti-phospholipid syndrome
ARF	Antibacterial response factor
BEVs	Bacterial extracellular vesicles
β_2_GP-1	β_2_ glycoprotein-1
cld1	Claudin1
EV	Extracellular vesicles
FABP2	Fatty acid binding protein 2
F/B	Firmicutes/Bacteroidetes
GALT	Gut-associated lymphoid tissue
GC	Glucocorticoid
GM	Gut microbiota
HCQh	Hydroxycloroquine
HDAC	Histone deacetylase
HHV	Human herpes virus
HSP	Heat-shock protein
IBD	Inflammatory bowel disease
IC	Immunocomplex
IFNy	Interferon gamma
ILC3	Innate lymphoid cells C3
LGS	Leaky gut syndrome
LN	Lupus nephritis
LPB	Lipopolysaccharide binding protein
LPS	Lipopolysaccharide
PAMP	Pathogen-associated molecular pattern
RA	Rheumatoid arthritis
RCT	Randomized controlled trial
RG	Ruminococcus Gnavus
RT	Roseburia Intestinalis
sCD14	Soluble CD14
SCFAs	Short-chain fatty acids
SFB	Segmented Filamentous Bacteria
SLE	Systemic lupus erythematosus
SLEDAI	Systemic lupus erythematosus Disease Activity Index
TLR	Toll-like receptor
Treg	T regulatory lymphocyte
ZO-1	Zonula Occludens 1
